# Prostate Cancer Lesions by Zone and Race: Does Multiparametric MRI Demonstrate Racial Difference in Prostate Cancer Lesions for African American Men?

**DOI:** 10.3390/curroncol28040212

**Published:** 2021-06-22

**Authors:** Christopher R. Koller, Jacob W. Greenberg, Thomas M. Shelton, William M. Hughes, Ganesh Sanekommu, Jonathan Silberstein, Louis S. Krane

**Affiliations:** 1 Department of Urology, Tulane University School of Medicine, New Orleans, LA 70112, USA; ckoller@tulane.edu (C.R.K.); jgreenberg@tulane.edu (J.W.G.); tshelton2@tulane.edu (T.M.S.); whughes3@tulane.edu (W.M.H.); gsanekommu@tulane.edu (G.S.); 2 Department of Genitourinary Oncology, Memorial Health System, Aventura, FL 33180, USA; jsilberstein@mhs.net; 3 Department of Urology, Southeastern Louisiana Veterans Health Care System, New Orleans, LA 70112, USA

**Keywords:** prostate cancer, MRI, race, lesions, outcomes

## Abstract

African American (AA) men have increased risk of prostate cancer diagnosis and mortality, but the cause remains unknown. MRI fusion improves diagnosis of localized prostate cancer, particularly in anterior lesions; however, cost and access are limited in a community practice setting. By utilizing a diverse cohort of veterans with equal access to care in a single payer system, we describe prostate cancer detection. We queried a prospectively maintained institutional review board-approved database of men undergoing prostate biopsy for untreated prostate cancer. We included all consecutive patients from October 2017 to February 2020. Statistical analysis including Kaplan–Meier Curves, Fisher’s exact test, and Forest plot was performed. From 246 consecutive patients, 166 were AA and 80 were non-AA. There were similar distributions of PSA, PSAD, and number of targetable lesions between the AA and non-AA cohort (*p* > 0.05 for all). We found no difference in location on MRI between race groups. There was similar cancer detection, focusing on anterior lesions and rate of positive Gleason grade (≥GG1) and clinically significant (≥GG2) cancer between cohorts. In a predominant AA cohort of veterans, we found similar distribution of location for MRI-targeted lesions, along with rates of tumor detection and aggressiveness of disease. In this single payer veteran population, we did not identify specific biologic differences inherent to tumor detection between AA and non-AA patients.

## 1. Introduction

Racial disparities in cancer outcomes continue to be a significant point of concern for the United States’ health system. In the urologic community, racial disparity is intensely present in prostate cancer outcomes. Despite many advances in prostate cancer diagnosis, risk stratification, and treatment options, African American (AA) patients have clinically worse outcomes when compared with non-AA cohorts. The incidence of prostate cancer is 60% greater in AA men than their white counterparts, and almost a twofold increase in prostate cancer-related deaths have been documented for AA men. AA men are also more likely to show disease progress while on active surveillance (AS) and throughout their treatment course after failing AS. Additionally, they have worse final pathologic outcomes, even when initially diagnosed with low-risk tumors [1,2].

Since multiparametric MRI (mpMRI) of the prostate with accompanying MRI fusion biopsy has become increasingly utilized, it has consistently demonstrated the ability to identify small clinically significant malignancies. MRI is relatively expensive, which may be why it is not commonly performed in patients outside of large academic centers; however, it has become more accessible in community practice, with a recent national survey demonstrating 38% of private urologists have implemented it in their practice as compared to 72% of academicians [3].

There has been significant under sampling of mpMRI and fusion biopsy data in both the veteran and AA communities, and a paucity of mpMRI data for AA patients makes understanding baseline characteristics and possible differences challenging. This overall scarcity of information makes comprehensive recommendations about AS inclusion criteria based on race limited, and the use of mpMRI may allow for a more sophisticated risk stratification. We sought to describe the findings of a large, primarily AA cohort of mpMRI data and its relationship to anatomic pathology from associated fusion biopsy data.

## 2. Materials and Methods

From October 2017 to February 2020, all veterans who qualified for a transrectal ultrasonography (TRUS) prostate biopsy were recommended to undergo a mpMRI for future MRI/TRUS fusion biopsy using the UroNav® (Phillips, Cambridge, MA, USA) fusion biopsy system at our institution. Patients were then enrolled into our prospective study, at a single-center institution, approved by the Southeast Louisiana Veterans Health Care System (SLVHCS) IRB. A total of 246 patients met our inclusion criteria and were registered into this study. The inclusion criteria were as follows: men presenting for detection or surveillance of localized prostate cancer with a life expectancy ≥10 years or men on an AS protocol for a previously diagnosed prostate cancer. All patients in this study underwent an MRI/TRUS fusion biopsy using the UroNav®. 

Prior to biopsy, the patient’s mpMRI was graded by a single abdominal radiologist at our institution using the Prostate Imaging Reporting and Data System (PIRADS). Biopsies were performed under the guidance of fellowship-trained urologic oncologists and took place in the urology clinic. Following biopsy, tissue cores were sent to the SLVHCS pathology department for evaluation. If concerns were raised by the pathologist, an additional pathologist’s opinion was obtained. If consensus on a patient’s Gleason grade (GG) was not agreed upon, pathology slides were sent for central reading at the Joint Pathology Center.

MRI imaging was paired with real-time TRUS imaging after the organ was outlined, registered, and fused per standard protocol. All targetable lesions identified on pre-biopsy MRI were sampled with at least two cores per lesion, starting with the highest risk lesion first and working down in PIRADS grades. Using the UroNav® software, we kept track of the previous samples to ensure adequate biopsy. After targeted lesions were biopsied, patients then underwent a standard 12-core prostate biopsy.

### Statistical Analysis

Patient clinical demographics, laboratory values, and biopsy profiles were evaluated using a statistical computational language called R 4.0.2 (Ann Arbor, MI, USA). Categorical comparisons were performed using Fisher’s exact tests. Continuous were assessed using a *t*-test, and granted data were normally distributed. Non-parametric continuous data were evaluated using the Mann–Whitney test. A Wilcoxon signed rank test was used to determine the differences in GG per PIRAD score. Receiver operating characteristics (ROCs) were generated using R package “pROC” [4]. Logistic regression was used to evaluate correlations between clinical features and GG. Prostate biopsy heatmaps were generated using R. All tests were two-tailed, using a significance cutoff of *p* < 0.05.

## 3. Results

A total of 246 patients (166 AA and 80 non-AA) met inclusion criteria and were entered into this study for analysis. Demographic characteristics throughout the groups were closely aligned; racial groups displayed statistically comparable median age and BMI and are described in Table 1. The median pre-biopsy prostate-specific antigen (PSA) was 6.34 (IQR, 4.29) for AA and 6.38 (interquartile range (IQR), 5.10) for non-AA (*p* = 0.74). Prostatic volumes calculated by MRI were also similar, 56g (IQR, 40.6) for AA and 50.3g (IQR, 33.3) non-AA (*p* = 0.16). The number of patients receiving a previous prostate biopsy was comparable between racial groups (*p* = 0.61). Additionally, AA patients had similar number of regions of interest (ROI) lesions on MRI compared to their non-AA cohort members. 

A multivariable logistic regression analysis was performed to determine the predictors associated with locating positive cancer (GG ≥ 1) on biopsy. Predictors of identifying GG ≥ 1 disease were decreasing MRI volume and any PIRADS-graded lesion with respective odds ratios of 0.97 (CI, 0.96–0.99) and 1.94 (CI, 1.34–2.87). A decreasing MRI volume and increasing PIRADS was associated with an increased rate of cancer (*p* < 0.001 both). Race was not found to be a predictor of locating GG ≥ 1 disease (*p* = 0.16) (Table 2).

**Figure 1:**
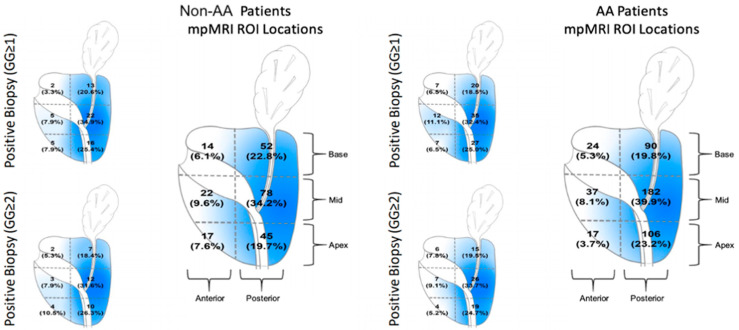
Heatmap illustrating ROI lesions found on mpMRI and positive detection for GG ≥ 1 and GG ≥ 2 between racial groups. Rates of ROI, GG ≥ 1, and GG ≥ 2 per prostate lobe location were statistically comparable between racial groups.

MRI PIRADS findings were then compared by lesion location between our racial groups (Figure 1). Both groups predominantly had lesions in the periphery of the prostatic, which is consistent with expected tumor distribution as described in the literature. The overall rate of positive cancer (GG ≥ 1) per ROI using the UroNav® software was found to be comparable between races (*p* = 0.38). This finding was mirrored with the rates of clinically significant (GG ≥ 2) cancer (*p* = 0.99). AA displayed no statistical difference in having less anterior MRI ROI lesions than non-AA patients (*p* = 0.064). Of the lesions sampled in the anterior prostate, AA and non-AA patients displayed comparable positive biopsy rates on both overall (GG ≥ 1) and clinically significant (GG ≥ 2) cancer per PIRADS. Additionally, when stratifying for PIRAD score at anterior, transitional, and peripheral zones, we found no differences in positive cancer rates. Moreover, when sorting on PIRAD score for each zone, we identified GG ≥ 2 at comparable rates (*p* > 0.05 for all these variables) (Table 3).

**Figure 2:**
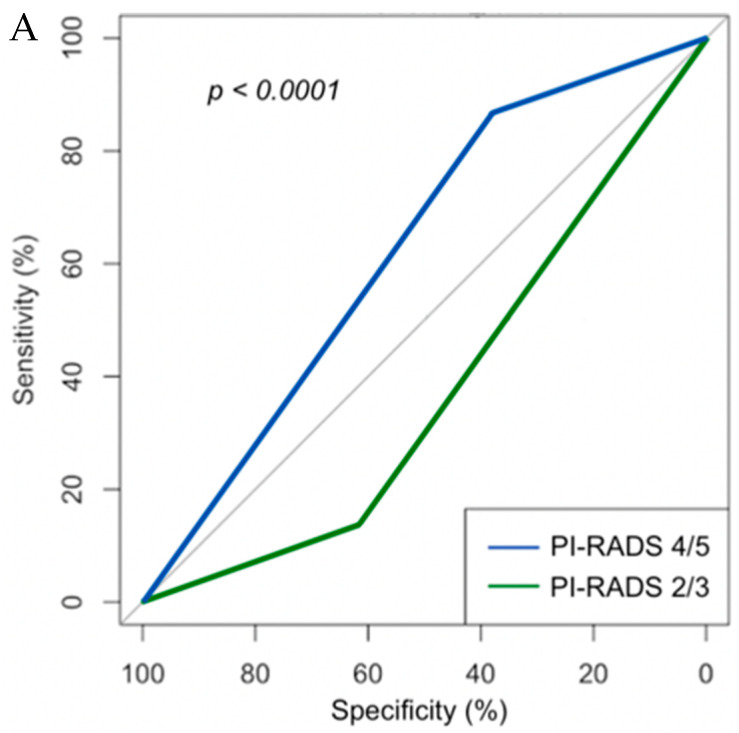

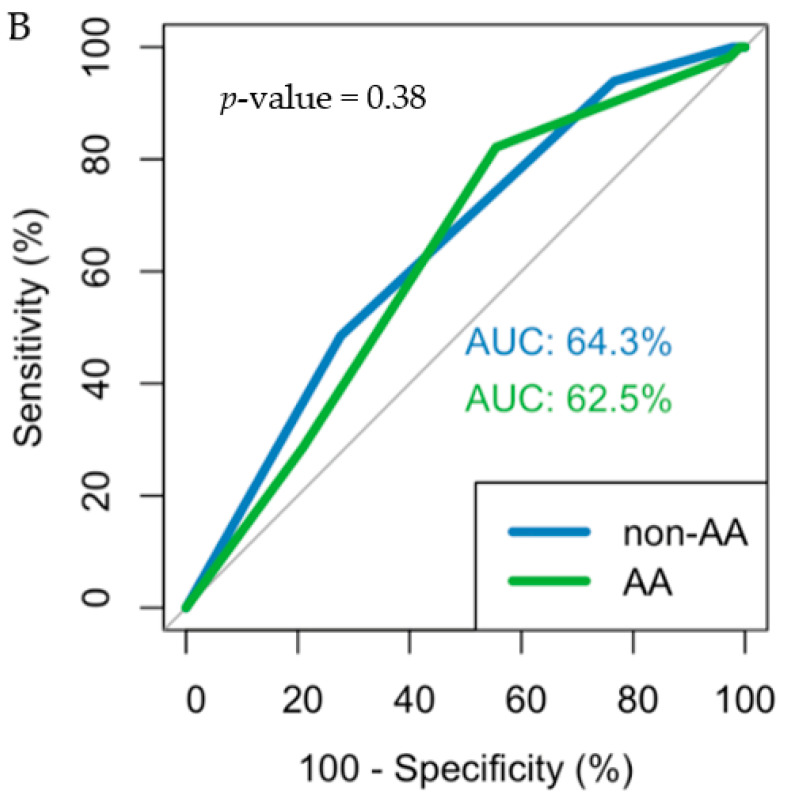
ROC regarding likelihood of clinically significant prostate cancer detection. (**A**) PI-RADS 4/5 were found to have statistically higher AUC. (**B**) When comparing racial groups, we found no statistical difference.

ROC curves were generated to assess the accuracy of mpMRI-guided biopsy between our racial groups. The outcome variable for these curves was set to locating GG ≥ 1 disease (Figure 2). To validate our finding, we compared the utility of PIRADS 4/5 to locate a cancerous lesion to that of PIRADS 2/3, regardless of race. GG was determined on the ROI-specific biopsies (Figure 2A). Our PIRADS 4/5 demonstrated an improved area under the curve (AUC) for locating cancer when compared to PIRADS 2/3 ROIs (*p* < 0.0001). ROC curved were then used to compare AUC between our racial groups (Figure 2B). AA patients showed statistically similar findings when compared to non-AA patients (*p* = 0.38).

## 4. Discussion

The use of mpMRI in combination with MRI/US fusion biopsy has increased in prevalence and has consistently illustrated many advantages for both the patient and the clinician. In working towards tools to help urologists detect clinically significant prostate cancer, MRI fusion has shown itself to be a modality to combat overdiagnosis and overtreatment [5,6,7,8,9]. Establishing which patients are the most likely to benefit from definitive therapies in the form of radical prostatectomy or radiotherapy is of primary concern. Of arguably equal concern, however, is determining which patients would not see any survival benefit from receiving treatment and to risk stratify those that may be appropriate candidates to place on an AS protocol. 

Up until this point, AA men evaluated and treated for prostate cancer have been unquestionably underrepresented in the mpMRI and fusion biopsy literature. AA men are 2.5 times more likely to die from prostate cancer and are much more likely to present with advanced disease at time of diagnosis [10]. For reference, the non-AA PCa-specific overall 10-year survival in NCCN low-risk patients was found to be 60% in a recent Canadian cohort [11,12]. There is an ongoing debate as to whether there is an inheritable difference that in and of itself increases AA men’s likelihood for poor prostate cancer outcomes versus socioeconomic factors playing a larger role [13].

Sundi et al. (2014) describe final pathology in a cohort of AA patients with very low and low-risk tumors, which demonstrated a higher prevalence of anterior cancer foci of high grade and larger tumors as compared to white men of similar demographics [14]. This may suggest that poor outcomes are a function of a yet-to-be established link between race and tumor biology. Our data, however, do not support this argument. In the largest AA mpMRI cohort to date, we found there is no statistical difference in PIRADS lesion location (notably in the anterior region), quantity of lesions, rate of cancer detection, or rate of positive biopsy. The similarity of the ROI’s location throughout the prostate in each cohort is well illustrated in the heatmap (Figure 1) [15].

This study provides strong evidence that there may in fact be no significant relationship to anterior prostate tumor location as it related to AA race. The broad implications of these findings support the idea that all men, regardless of race, can be evaluated accurately with MRI fusion biopsy technology. Furthermore, our evidence supports that AS can be safely considered in this population without fear of imperceivable cancer in the anterior of the prostate in AA men, leading to worse oncologic outcomes [16]. These findings may later be important to the advances in diagnostics in combination with genetic and biomarker technologies, as well as for treatment options such as focal therapies. 

Our study is not without its limitations. First, this is a single institutional database, and any collective and unidentified error may be due to larger systemic procedures. Secondly, our cancer detection rate was lower than expected, while the focus of ongoing efforts for quality improvement at this rate appears substandard. However, despite limited success in identifying clinically significant cancer, both AA and non-AA cohorts likely indicated no variance in race. Furthermore, we believe this cohort, without dedicated fellowship-trained MRI prostate radiologists, may reflect a more accurate depiction of “real-life experience” in MRI fusion outside of centers of excellence with dedicated prostate MRI teams. Of note some location subsets had few patients, sometimes less than 5 per group. However, the overall comparison of importance to this study, anterior vs. posterior lobe, were comparable between racial groups. 

Additional considerations are related to demographics of the patient pool itself. The genetics of this patient pool is unknown, and the lineage of these men may be important. There has been evidence suggesting that outcomes for men of West African descent are different in Caribbean Islands and the United Kingdom as compared to the United States [17]. Louisiana was an active participant in the transatlantic slave trade, and as Odedina et al. discussed, most slaves of West African populations were from Benin, Nigeria, Ghana, Gambia, and Senegal. Historical records compiled from The Department of Cultural Development of the State of Louisiana illustrates that the majority of slaves transported to Louisiana in the 1700s to 1800s were largely from Senegambia (a region lying between Senegal and Gambia) as well as the Gold Coast and Benin [18]. To what extent this is generalizable to AA men may be difficult to answer.

Lastly, veterans themselves may have unique risk factors that would preclude generalizability to the rest of the non-veteran populations. There is evidence that those who utilize Veterans Affairs facilities for their care have different socioeconomic and medical problems than veterans who opt for community medical care; it would be reasonable to propose that there are differences between veterans and non-veterans as well [19,20,21].

Due to limitations in referral patterns and PSA testing, it is possible that there could be an underlying variable that is not well characterized, causing poorer inclusion for patients that could potentiate missed diagnoses of high-risk tumors. Our data support the notion that disparities in prostate cancer outcomes in AA patients is not due to these patients having a higher likelihood of tumor foci in the anterior prostate and is likely the composite of other elements of their tumor biology in conjunction with social determinants of health.

## 5. Conclusions

Inferior outcomes for AA patients do not appear to be due to inability to locate or identify lesions on MRI. Increased adoption of this technology may ultimately improve cancer detection and long-term outcomes for AA patients with clinically localized prostate malignancy.

## Figures and Tables

**Table 1:** Patient cohort demographic and ROI characteristics.

Demographics (IQR)	Total	AA	Non-AA	*p*-Value
Number (%)	246 (100%)	166 (67.5%)	80 (32.5%)	---
Median age	68.05 (7.8)	68.0 (7.08)	68.2 (8.96)	0.87
Median BMI	28.9 (6.59)	29.1 (6.71)	28.4 (6.48)	0.53
Median Bx PSA (ng/ml)	6.37 (4.69)	6.34 (4.29)	6.38 (5.10)	0.74
Median PSA density	0.13 (0.12)	0.13 (0.14)	0.14 (0.10)	0.89
Median testosterone	3.12 (1.16)	3.19 (1.33)	2.89 (1.13)	0.15
Median prostate volume TRUS	46.8 (±29.4)	48.3 (33.0)	44.0 (23.8)	0.58
Median prostate volume MRI	53.8 (40.1)	56.0 (±40.6)	50.3 (±33.3)	0.16
Previous Bx (%)	---	---	---	---
- Yes (TRBx)	199 (80.9%)	136 (82%)	63 (79%)	---
- No	47 (19.1%)	30 (18%)	17 (21%)	0.61
Median total ROI count (%)	---	---	---	---
- ROI ≥ 1	246 (100%)	166 (100%)	80 (100%)	---
- ROI ≥ 2	180 (73.2%)	121 (72.9%)	59 (73.8)	---
- ROI ≥ 3	87 (35.3%)	58 (34.9%)	29 (36.3%)	---
- ROI ≥ 4	24 (9.76%)	15 (9.04%)	9 (11.3%)	---
- ROI ≥ 5	1 (0.41%)	0 (0%)	1 (1.25%)	---

**Table 2:** Logistical regression of demographic characteristics and likelihood of cancer detection.

Variable	OR (95% CI)	*p*-Value
Age	1.03 (0.98–1.08)	0.22
BMI	0.98 (0.93–1.03)	0.38
Race	1.05 (0.58–1.95)	0.16
PSA	1.03 (0.99–1.12)	0.35
MRI volume	0.97 (0.96–0.99)	<0.001
PSAD	0.60 (0.01–1.47)	0.26
PI-RADS score	1.94 (1.34–2.87)	<0.001

**Table 3:** Overall analysis of racial composition, PIRADS location, and prostate cancer detection. The number of positive ROI biopsies with Gleason grade ≥1 and ≥2 were statistically comparable between racial groups.

ROI Location	AA		Non-AA		*p*-Value
	*n* Positive	*n* Negative	*n* Positive	*n* Negative	
GG ≥ 1					
Overall (%)	76 (23%)	260 (77%)	45 (26%)	126 (74%)	0.38
Anterior lesion (%)	---	---	---	---	0.83
- PI-RADS 3	5 (6%)	26 (10%)	2 (5%)	7 (6%)	0.64
- PI-RADS 4	5 (6%)	9 (3.5%)	4 (9%)	22 (17%)	0.23
- PI-RADS 5	9 (12%)	9 (3.5%)	6 (13%)	4 (3%)	0.71
Transitional zone (%)	---	---	---	---	0.99
- PI-RADS 3	4 (5%)	18 (7%)	1 (2%)	6 (5%)	0.99
- PI-RADS 4	4 (5%)	14 (5%)	0 (0%)	6 (5%)	0.54
- PI-RADS 5	0 (0%)	6 (2%)	2 (4%)	5 (4%)	0.46
Peripheral zone (%)	---	---	---	---	0.21
- PI-RADS 3	18 (24%)	106 (41%)	3 (7%)	31 (25%)	0.57
- PI-RADS 4	25 (33%)	56 (22%)	19 (42%)	33 (26%)	0.57
- PI-RADS 5	6 (8%)	16 (6%)	8 (18%)	12 (9%)	0.52
GG ≥ 2					
Overall (%)	53 (16%)	283 (84%)	27 (16%)	144 (84%)	0.99
Anterior lesion (%)	---	---	---	---	0.99
- PI-RADS 3	2 (4%)	29 (11%)	0 (0%)	9 (6%)	0.99
- PI-RADS 4	3 (6%)	11 (4%)	2 (7.5%)	24 (17%)	0.32
- PI-RADS 5	7 (13%)	11 (4%)	6 (22%)	4 (3%)	0.43
Transitional zone (%)	---	---	---	---	0.26
- PI-RADS 3	4 (7.5%)	18 (6%)	1 (4%)	6 (4%)	0.99
- PI-RADS 4	4 (7.5%)	14 (5%)	0 (0%)	6 (4%)	0.54
- PI-RADS 5	0 (0%)	6 (2%)	0 (0%)	7 (5%)	0.99
Peripheral zone (%)	---	---	---	---	0.63
- PI-RADS 3	10 (19%)	114 (40%)	2 (7.5%)	32 (22%)	0.99
- PI-RADS 4	18 (34%)	63 (22%)	10 (37%)	42 (29%)	0.83
- PI-RADS 5	5 (9%)	17 (6%)	6 (22%)	14 (10%)	0.73

## Data Availability

Data available on request due to privacy restrictions. The data presented in this study are available on request from the corresponding author. The data are not publicly available due to concerns over exposing protected health information.
